# Association of *ACTN3* R577X Polymorphism with Elite Basketball Player Status and Training Responses

**DOI:** 10.3390/genes14061190

**Published:** 2023-05-29

**Authors:** Berkay Demirci, Celal Bulgay, Halil İbrahim Ceylan, Mehmet Ertuğrul Öztürk, Deniz Öztürk, Hasan Huseyin Kazan, Mehmet Ali Ergun, Mesut Cerit, Ekaterina A. Semenova, Andrey K. Larin, Edward V. Generozov, Ildus I. Ahmetov, Ladislav Cepicka

**Affiliations:** 1 Sports Science Faculty, Lokman Hekim University, 06510 Ankara, Türkiye; 2 Sports Science Faculty, Bingol University, 12000 Bingol, Türkiye; 3 Physical Education and Sports Teaching Department, Kazim Karabekir Faculty of Education, Ataturk University, 25240 Erzurum, Türkiye; 4 Vocational School of Health Services, Ataturk University, 25240 Erzurum, Türkiye; 5 Department of Medical Genetics, Near East University, Nicosia 99138, Cyprus; 6 DESAM Institute, Near East University, Nicosia 99138, Cyprus; 7 Department of Medical Genetics, Faculty of Medicine, Gazi University, 06560 Ankara, Türkiye; 8 Department of Molecular Biology and Genetics, Federal Research and Clinical Center of Physical-Chemical Medicine of Federal Medical Biological Agency, 119435 Moscow, Russia; 9 Research Institute of Physical Culture and Sport, Volga Region State University of Physical Culture, Sport and Tourism, 420138 Kazan, Russia; 10 Laboratory of Genetics of Aging and Longevity, Kazan State Medical University, 420012 Kazan, Russia; 11 Department of Physical Education, Plekhanov Russian University of Economics, 115093 Moscow, Russia; 12 Research Institute for Sport and Exercise Sciences, Liverpool John Moores University, Liverpool L3 5AF, UK; 13 Department of Physical Education and Sport, Faculty of Education, University of West Bohemia, 30100 Pilsen, Czech Republic

**Keywords:** physical performance, athletes, Yo-Yo IR 2, rs1815739, genotype

## Abstract

The α-actinin-3 (*ACTN3*) gene rs1815739 (C/T, R577X) polymorphism is a variant frequently associated with athletic performance among different populations. However, there is limited research on the impact of this variant on athlete status and physical performance in basketball players. Therefore, the aim of this study was twofold: (1) to determine the association of *ACTN3* rs1815739 polymorphism with changes in physical performance in response to six weeks of training in elite basketball players using 30 m sprint and Yo-Yo Intermittent Recovery Test Level 2 (IR 2) tests, and (2) to compare *ACTN3* genotype and allelic frequencies between elite basketball players and controls. The study included a total of 363 individuals, comprising 101 elite basketball players and 262 sedentary individuals. Genomic DNA was isolated from oral epithelial cells or leukocytes, and genotyping was performed by real-time PCR using KASP genotyping method or by microarray analysis. We found that the frequency of the *ACTN3* rs1815739 XX genotype was significantly lower in basketball players compared to controls (10.9 vs. 21.4%, *p* = 0.023), suggesting that RR/RX genotypes were more favorable for playing basketball. Statistically significant (*p* = 0.045) changes were observed in Yo-Yo IRT 2 performance measurement tests in basketball players with the RR genotype only. In conclusion, our findings suggest that the carriage of the *ACTN3* rs1815739 R allele may confer an advantage in basketball.

## 1. Introduction

Based on their years of experience and observations, sports scientists and coaches have suggested that there were significant differences in the development of physical performance among individuals. They have also observed that exercise loads triggered personalized behavioral changes, significantly contributing to the performance and a healthy metabolism. The human metabolism is influenced by the composition of many different systems. Recent research has shown that individual differences in genetic background can affect responses to training loads, the effectiveness of ergogenic aids, recovery rates, calorie needs, and the risk of injury. In addition, it is anticipated that similar research findings could provide a stronger basis for individualized training methods. Nevertheless, the information obtained from genetic tests is still insufficient to provide optimal performance and training variables in the individual level in sport genetics [1].

Recent studies on athletic performance have focused on genetic variants that significantly contribute to the individual performance [2,3]. Individual characteristics such as aerobic and anaerobic endurance, speed, strength, distribution of muscle fiber types, blood pressure, testosterone levels, intra- and intermuscular coordination, and motivation are highly influenced by genes [4,5,6,7]. Researchers in the field of sports genetics have shown that a person could up to a certain level reach optimum performance through the correct and systematic application of training, and that the athlete’s performance level was limited by the genetic background [8,9,10,11,12,13]. Therefore, it is predicted that performing genetic tests in addition to sport-specific field and laboratory performance tests at the elite level could be used to regulate athletes’ performance and training loads for higher athletic performance [1,14].

In skeletal muscle, the mechanism consists of repeated contractions of organized arrays of thin filaments containing actin and thick filaments including myosin or sarcomeres. The Z-lines are dense three-dimensional structures that run perpendicular to the myofibrils and anchor the thin filaments. Sarcomeric α-actinins serve as the main protein component of Z-lines and cross-linking actin thin filaments to maintain the ordered myofibril array. In mammals, the α-actinin-3 (*ACTN3*) genes encode for skeletal muscle α-actinins [15]. A-actinin-3 is only found in type II (fast and glycolytic) muscle fibers [16]. The specialized expression of *ACTN3* in fast muscle fibers can also affect skeletal muscle metabolism or fiber type specification. Therefore, the *ACTN3* gene has become a popular marker influencing exercises. A C>T transition at the codon 577 of the *ACTN3* gene is described as a common genetic variation that results in a stop codon (X) leading to the production of a nonfunctional gene product [17,18,19]. In individuals of European descent, less than one-third of the population have two copies of the functional protein, the so-called arginine I allele (RR genotype), while over half of the population have one copy of each allele (RX genotype) [20]. In this context, the absence of α-actinin-3 in the fast fibers of skeletal muscle may affect functional properties and cause variations in muscular functions within the general population.

It is likely that α-actinin-3 deficiency impairs the performance of fast-twitch skeletal muscle fibers both in elite athletes and nonathletic populations. Many studies have proved a negative correlation between the rs1815739 XX genotype of the *ACTN3* gene and elite speed activities [21,22,23]. Furthermore, XX genotypes were observed to cause lower levels of basic muscle strength compared to RX and RR ones [24,25]. All of these studies support the idea that *ACTN3* deficiency inhibits the performance of fast glycolytic muscle fibers responsible for explosive muscle contractions.

Data suggest that the presence of α-actinin-3 is necessary for optimal fast-twitch muscle fibers or rapid muscle contraction in power athletes, while its absence may provide some advantages for athletes engaged in endurance-type activities [2,26]. Many studies have supported the conclusion that the R577X polymorphism had a clear biochemical effect of complete elimination of the production of functional protein, being consistent with the negative effects of α-actinin-3 deficiency on sprint and power-based sports [6,27,28].

Although the involvement of *ACTN3* rs1815739 polymorphism has widely been studied in diverse sport branches and parameters, the association of this variant with athletic parameters in basketball players has limitedly been reported. Hence, the purpose of the present study was twofold: (1) to determine the association of *ACTN3* rs1815739 polymorphism with changes in physical performance in response to six weeks of training in elite basketball players using 30 m sprint and Yo-Yo Intermittent Recovery Test Level 2 (IR 2) tests, and (2) to compare *ACTN3* genotypic and allelic frequencies between elite basketball players and controls. We believe that this study on elite athletes will contribute to the literature, and the findings will pave the way for similar studies in the future.

## 2. Materials and Methods

### 2.1. Ethical Approval

The study was carried out in accordance with the Declaration of Helsinki, and approval was obtained from the Lokman Hekim University Non-Interventional Clinical Research Ethics Committee with decision number 2022-17/1 and the Ethics Committee of the Federal Research and Clinical Center of Physical-Chemical Medicine of the Federal Medical and Biological Agency of Russia (Approval number 2017/04).

### 2.2. Participants

The present study was conducted with the participation of professional basketball players from Turkey (*n* = 20) and Russia (*n* = 81). The control group consisted of Turkish physically inactive students from the Faculty of Sports Sciences (*n* = 47) and 215 Russian sedentary individuals. Characteristics of study participants are shown in Table 1. Performance tests were applied to the Turkish participants only, and Russian participants were involved in the case-control study. The participants were informed about the measurement procedure, and the informed consent forms were signed by the participants before the measurements. Samples for DNA isolation were obtained by oral swabbing or as peripheral blood.

### 2.3. Evaluation Methods for Exercise and Performance

The professional Turkish athletes (*n* = 20) participated in training six days a week for approximately 60–75 min per day. The microcycle of the training included two days of double-training sessions with a focus on anaerobic load and four days of single-training sessions, totaling eight sessions per week (approximately 480 min per week) of training.

All of the professional athletes who participated in this study were familiar with the test procedures due to the training and evaluation practices at the club where they registered. Detailed information was provided to the Sports Science Faculty students regarding the protocol for administering the tests, and they were given the opportunity to practice several times before the test. Approximately 7–8 min of warm-up running and 8–10 min of stretching exercises were applied to the lower and upper extremities after the tests for all participants. Data on the players’ recovery skills after high-intensity runs were collected using the Yo-Yo IR 2 test. In addition, data on anaerobic power performance were determined by the 30 m speed using the Witty Speed photocell device.

### 2.4. Data Collection Tools

#### 2.4.1. Yo-Yo IR 2 Test

The Yo-Yo IR test is one of the priority methods for assessing fitness in sports sciences. It allows for evaluating the capacity of performing high-intensity exercises repeatedly by utilizing both aerobic and anaerobic energy systems at the same time. The test is frequently used in various team sports to evaluate players’ ability to participate in high-intensity activities continuously due to its specificity and practicality. The Yo-Yo IR 2 test consists of shuttle runs of increasing speeds of 40 (2 × 20) meters which are separated by active recovery periods of 10 s (controlled by sound signals from a compact disk player). Participants continue to run until they can no longer keep up with the current speed. The distance at which the participants stop the run due to failing to reach the marked point twice in a row determines the test’s result. The test takes 5–15 min and measures an individual’s ability to perform high-intensity exercise repetitions with a high anaerobic energy contribution, assuming that the individual is well trained. Since its introduction to the general public [29], the Yo-Yo IR 2 test has been widely used in team sports [30]. In the present study, the Yo-Yo IR 2 test was performed on all participants as the method summarized above.

#### 2.4.2. 30 m Sprint Test

The 30 m sprint test of the players was performed by the photosensor Witty Speed, Microgate Equipment, ITA device. Basketball players who stood 0.5 m away from the starting line executed two sets of 30 m sprint runs. Sprint tests were performed on a closed running track to prevent the effects of weather conditions. A five-minute rest period was given between the two attempts, and the fastest time was recorded for analysis.

#### 2.4.3. *ACTN3* rs1815739 Polymorphism Analysis

The molecular tests in Turkish athletes and controls were carried out at the Damagen Genetic Diagnosis Center in Ankara. Genomic DNAs from oral swabs were isolated using the Buccalyse DNA Extraction Kit (Isohelix, UK), according to the supplier’s instructions. The DNA concentration was determined using a NanoDrop spectrophotometer (Thermo Fisher Scientific, Waltham, MA, USA). The genotyping of the single-nucleotide polymorphism (SNP) was performed using the KASP genotyping method (LGC Genomics, Beverly, CA, USA). In brief, the KASP assay mixture contained three oligos that were assay-specific and unlabeled, including two allele-specific forward and a common reverse primer. The KASP Master Mix (2X) was purchased as a ready-to-use solution containing the universal fluorescent dyes FAM and VIC in the presence of ROX passive dye. After adding the SNP-specific KASP primers and the universal KASP Master mixture to the DNA samples, PCR reaction was performed on the 7500 Real-time PCR System (Applied Biosystems, Foster City, CA, USA) device. Then, fluorescence readings were taken, and the obtained data were analyzed according to the previous reports [31].

Molecular genetic analysis in Russian subjects was performed with DNA samples obtained from peripheral blood. Four mL of venous blood were collected in tubes containing EDTA (Vacuette EDTA tubes; Greiner Bio-One, Kremsmunster, Austria). DNA extraction and purification were performed using a commercial kit according to the manufacturer’s instructions (Technoclon, Moscow, Russia). HumanOmniExpressBeadChips (Illumina Inc., San Diego, CA, USA) were used in the microarray analysis to genotype *ACTN3* rs1815739 polymorphism.

### 2.5. Statistical Analysis

Statistical analyses were performed using the Statistical Package for Social Sciences (SPSS 25.0) program. Descriptive statistics including number, percentage, mean, and standard deviation were primarily used to evaluate the data. According to the results of the Kolmogorov–Smirnov and Shapiro–Wilk tests, it was determined that the data displayed a normal distribution. In this context, the Paired Samples *t*-test was used to examine the differences between the pre- and post-test performances of the athletes and the unpaired *t*-test was used to examine the differences based on the variables between the groups. Genotype and allele frequencies were calculated and Hardy–Weinberg equilibrium (HWE), chi-square (χ^2^), and Fisher’s exact tests were used to evaluate the differences in allelic or genotype frequencies between athletes and controls. Hypotheses were tested at a significance level of *p* < 0.05 with a 95% confidence interval.

## 3. Results

There were no differences in the allelic frequencies between Turkish and Russian controls (frequency of allele R: 56.4% in Turkish and 57.0% in Russian controls). There were no significant differences in *ACTN3* genotype and allele frequencies between males and females amongst athletes and controls. No effect of age on *ACTN3* genotype and allele frequencies has been found (*p* > 0.05). We, therefore, felt justified to combine the data of Turkish and Russian individuals. 

Both in 101 basketball players (*p* = 0.273) and 262 controls (*p* = 0.07), the *ACTN3* gene rs1815739 polymorphism met Hardy–Weinberg expectations. The frequency of the *ACTN3* XX genotype was significantly lower in basketball players compared to controls (10.9 vs. 21.4%, *p* = 0.023; Table 2), suggesting that RR and RX genotypes are more favorable for playing basketball.

As shown in Table 3, statistically significant differences were observed in Yo-Yo IRT 2 performance measurement tests within the basketball players with the RR genotype (*p* = 0.045) only, and the control group with RR, RX, and XX genotypes (*p* = 0.034, *p* = 0.002, and *p* = 0.008, respectively) in terms of pre- and post-test results. Additionally, in the 30 m sprint performances of the basketball and control groups specified in Table 3, no statistically significant results were found for pre- and post-test within each group (*p* > 0.05). 

Importantly, when the groups were compared in terms of pre- and post-test separately, significant differences were observed in the Yo-Yo IRT 2 performance measurement for the frequencies of RX and XX genotypes (pre-test; *p* = 0.06 and *p* = 0.010, respectively), XX genotypes (post-test; *p* = 0.018), and in the 30 m sprint test for RR, RX, and XX genotypes (pre-test; *p* = 0.001, *p* = 0.001 and *p* = 0.001, post-test; *p* = 0.001, *p* = 0.001 and *p* = 0.001, respectively) (Table 4).

## 4. Discussion

The main finding of our study was that the *ACTN3* XX genotype was underrepresented in elite basketball players, and the most significant changes in physical performance in response to training were observed in basketball players with the RR genotype, suggesting that for playing basketball it is beneficial to have power-increasing genotypes (i.e., *ACTN3* RR/RX).

It is generally accepted that biomotor abilities of both elite and non-elite athletes could be linked to genetic variation [32,33,34,35,36,37]. For mechanistic explanation of the biomotor abilities, many studies have indicated that the *ACTN3* gene was a highly critical candidate gene whose product affected the athletic performance in elite or sub-elite athletes [38,39,40,41]. The decisive role of *ACTN3* in high-intensity muscle contractions indicates that *ACTN3* R577X polymorphism is the variant linked to the individual performance differentiations in the basketball players [42]. Considering the fast and explosive muscle power required by basketball players and the key role of α-actinin-3 in muscle strength and power, it could be regarded that examination of *ACTN3* rs1815739 polymorphism may be helpful to at least partially explain the differences in the personal performances in basketball.

Several studies have described that the *ACTN3* R577X polymorphism was one of the variants influencing the muscle fiber types [2,43]. These studies have reported that elite power athletes with the RR genotype had a higher proportion of fast-twitch muscle fibers. Additionally, it has been emphasized that at least one copy of the allele C was required for the production of the protein that was sufficient for successful performance in elite-level power sports [43,44]. In contrast, individuals with the XX genotype have been found to have lower levels of fast-twitch muscle fibers [2,18,21,22,43,45,46,47,48,49], low testosterone release levels [6] and muscle strength [18,50], and higher levels of aerobic endurance capacity [51]. Therefore, identification of the allelic distributions of this polymorphism could be proper to evaluate the individual skills and clarify the most convenient training programs. Nonetheless, the results of studies would sometimes be controversial. While some studies have found that α-actinin-3 deficiency had a negative impact on elite sprint/power performance, it has been determined that it did not negatively correlate with muscle strength and anaerobic performance in other studies [25,52,53]. Thus, further association studies are needed for conclusive interpretations. Accordingly, the present study evaluated the differences in individual performances observed as a result of physiological interactions arising from the *ACTN3* rs1815739 gene variants in professional basketball players.

Similar to the studies reporting the allelic distribution of the rs1815739 polymorphism in elite athletes (including basketball players) and responses to exercise [54,55,56], our results underlined significantly higher frequency of the RR and RX genotypes in basketball players and better responses to exercise in RR genotype carriers in basketball players compared to controls. Nonetheless, contrary to our findings, some of the previous studies have reported that the *ACTN3* R577X polymorphism had no effect on sprint/power parameters, and that there was no statistically significant relationship between the rs1815739 polymorphism and athlete status or elite athletic performance [19,24,57,58,59,60]. On the other hand, Yang and his colleagues (2003) reported that none of the speed-power-oriented athletes observed in the Olympic Games were carriers of the XX genotype, and other studies have found that individuals with the allele R, having a high proportion of type II muscle fibers and muscle strength, exhibited higher maximal strength and greater muscle volume after nine weeks of lower-limb resistance training [43,61,62,63,64].

Basketball players cover an average distance of 6000 to 7500 m during a match, and approximately 200 out of the total 1100 activities are reported to be high-intensity movements [65,66,67]. In addition, it has been reported that short-term (2–6 s) maximal speed runs were repeated at every 30 s [68], and players performed approximately 40 to 60 jumps requiring high explosive power during a match [69]. Based on the information provided, it is understood that the majority of the energy required in basketball and similar disciplines is supplied by the adenosine triphosphate–creatine phosphate (ATP–CP) pathway and anaerobic glycolysis. Considering the long duration of a basketball match, aerobic endurance and short-term high-intensity explosive activities are quite important [30,32,70,71]. During these activities, energy is provided from both aerobic and anaerobic metabolisms. However, the main contribution of aerobic metabolism is the replenishment process of ATP sources depleted during high-intensity short-term loads during the competition [66,72]. During competitions, short rest intervals immediately after high-intensity efforts (9–12 s) allow for the replenishment of ATP–CP sources in muscles through the aerobic system. On the other hand, in mutual attacks and defense struggles where the intensity of loading is lower, anaerobic glycolysis (30 s to 2–3 min) becomes dominant [73,74]. Basketball players need to have well-developed anaerobic and aerobic fitness components (strength, speed, and endurance) in order to overcome the negative effects of high-intensity efforts during the short recovery phases of the play [65,75].

In our study, it was observed that there were significant differences in Yo-Yo IR 2 test performance only in the RR genotype within the players, while statistically significant differences were observed in all three genotypes within the control group when the pre- and post-tests were compared. Nevertheless, no statistically significant results were observed in the 30 m sprint test performance within both groups. Contrary to our findings, in a study conducted on football players, it was found that players with the XX genotype had significantly higher VO_2max_ values compared to those with the RR genotype group. Furthermore, compared to players with the XX genotype, players with the RR genotype were found to have significantly lower times at 10, 20, and 30 m distances [76] with higher acute recovery levels [77].

In another study that overlaps with our findings, the results showed that the percentage of RR genotype and the distribution of allele C were significantly higher among professional basketball players compared to other variables [78,79]. On the contrary, in a study by Garatachea and colleagues [80] that examined the relationship between the *ACTN3* R577X polymorphism and explosive power of leg muscles in elite basketball players, no association was found. Additionally, no significant differences were reported in terms of genotype distributions between the control group and the players [80]. Moreover, according to Coelho and colleagues (2016), *ACTN3* R577X is not an ideal genetic marker for identifying a talented football player, and besides physical fitness that affects high performance in team sports, technical and tactical skills are also important [32]. Another study conducted by Lima et al. [54] evaluated the rs1815739 polymorphism in elite basketball players in terms of player positions. As a result, an association was found between the RR genotype and the power forward and center positions. In addition, the RX genotype was shown to be highly associated with shooting guard and small forward positions [54]. In line with our data, these results confirm that the RR genotype may associate with explosive muscle strength and is perfectly linked to position requirements.

Critically, when Yo-Yo IR 2 and 30 m sprint test results were analyzed where the pre- and post-test were separately evaluated between the basketball players and control groups, the scores were figured out to be better in the players, as expected. Although there were significances for all genotypes for the 30 m test, the frequencies of the genotypes RX for pre-test and XX for pre- and post-test were significantly deviated between the basketball players and control groups. The higher degrees of the players in these tests could be explained by the players’ familiarity with the performance tests as well as their well-balanced sport experiences, diets, and psychologies.

In professional team sports, one of the most important factors affecting the outcome along with technical and tactical applications, motivation, and fitness levels are also fundamental ones. Still, the interactions revealed by genetic variants that manifest athletic ability provide important clues for training practices, including the scope and intensity of training, loading, recovery, and performance at the elite level in athletes who perform high-level effort.

To cumulatively understand the possible associations, further studies pointing to the alterations in the motor skill by R577X are needed. Moreover, the present study has some limitations including a limited sample size of athletes involved in the training response study, the inability to perform biochemical analyses, and the lack of control over variables such as epigenetics, limitations with the performance tests, and environment. In future research, we assume that more comprehensive information about this polymorphism could be provided by eliminating these limitations with larger sample groups and a higher number of informative performance tests in different disciplines.

## 5. Conclusions

In conclusion, our findings suggest that the carriage of the *ACTN3* rs1815739 allele R may confer an advantage in basketball. Still, further studies involving a higher number of participants and multigenetic approaches are needed.

## Figures and Tables

**Table 1:** Characteristics of study participants.

Group	*n*	Age	Sex (Males/Females)
Turkish basketball players	20	26.5 ± 8.0	20/0
Russian basketball players	81	29.2 ± 3.9	40/41
Turkish controls	47	23.8 ± 4.2	47/0
Russian controls	215	44.8 ± 4.4	160/55

**Table 2:** Comparison of genotype and allele frequencies of *ACTN3* rs1815739 polymorphism between basketball players and controls.

Group	*n*	*ACTN3* rs1815739 Genotypes			*p* (XX)	Allele, %		*p*
		RR	RX	XX		R	X	
Basketball players	101	38 (37.6%)	52 (51.5%)	11 (10.9%)	0.023 *	63.4	36.6	0.13
Controls	262	92 (35.1%)	114 (43.5%)	56 (21.4%)		56.9	43.1	

* *p* < 0.05, statistically significant differences in the XX genotype frequency between athletes and controls.

**Table 3:** Investigation of Yo-Yo IRT 2 and 30 m performance differences according to genotypes in basketball players and controls.

Group	Variable	Genotype	N	M ± SD		*p*
Basketball	Yo-Yo IRT 2 (m)	RR	6	Pre-test	670 ± 96	0.045 *
				Post-test	700 ± 94	
		RX	10	Pre-test	694 ± 90	0.096
				Post-test	718 ± 73	
		XX	4	Pre-test	800 ± 170	0.769
				Post-test	810 ± 116	
Controls	Yo-Yo IRT 2 (m)	RR	16	Pre-test	562 ± 164	0.034 *
				Post-test	617 ± 157	
		RX	21	Pre-test	496 ± 199	0.002 *
				Post-test	624 ± 233	
		XX	10	Pre-test	410 ± 229	0.008 *
				Post-test	480 ± 225	
Basketball	30 m	RR	6	Pre-test	4 ± 0	0.328
				Post-test	4 ± 0	
		RX	10	Pre-test	4 ± 0	0.600
				Post-test	4 ± 0	
		XX	4	Pre-test	4 ± 0	0.947
				Post-test	4 ± 0	
Controls	30 m	RR	16	Pre-test	5 ± 0	0.602
				Post-test	5 ± 0	
		RX	21	Pre-test	5 ± 0	0.117
				Post-test	5 ± 0	
		XX	10	Pre-test	5 ± 0	0.817
				Post-test	5 ± 0	

* *p* < 0.05, statistically significant changes within each genotype group.

**Table 4:** Investigation of 30 m and Yo-Yo IRT 2 performance differences according to genotype frequencies between the basketball and control groups.

Genotype	Variable	Group	N	Test	M ± SD	*p*
RR	Yo-Yo IR 2 (m)	Players	6	Pre-test	670 ± 96	0.151
		Controls	16		562 ± 164	
		Players	6	Post-test	700 ± 94	0.247
		Controls	16		617 ± 157	
RX	Yo-Yo IR 2 (m)	Players	10	Pre-test	694 ± 90	0.006 *
		Controls	21		496 ± 199	
		Players	10	Post-test	718 ± 73	0.231
		Controls	21		624 ± 233	
XX	Yo-Yo IR 2 (m)	Players	4	Pre-test	800 ± 170	0.010 *
		Controls	10		410 ± 229	
		Players	4	Post-test	810 ± 116	0.018 *
		Controls	10		480 ± 225	
RR	30 m	Players	6	Pre-test	4 ± 0	0.001 *
		Controls	16		5 ± 0	
		Players	6	Post-test	4 ± 0	0.001 *
		Controls	16		5 ± 0	
RX	30 m	Players	10	Pre-test	4 ± 0	0.001 *
		Controls	21		5 ± 0	
		Players	10	Post-test	4 ± 0	0.001 *
		Controls	21		5 ± 0	
XX	30 m	Players	4	Pre-test	4 ± 0	0.001 *
		Controls	10		5 ± 0	
		Players	4	Post-test	4 ± 0	0.001 *
		Controls	10		5 ± 00	

* *p* < 0.05, statistically significant differences within each genotype group.

## Data Availability

The data presented in this study are available on request from the corresponding authors.
